# Resilience and Concussion Recovery in Minority Women: Promoting Health Equity

**DOI:** 10.1089/neur.2024.0075

**Published:** 2024-10-09

**Authors:** Leon Ruiter-Lopez, Jack K. Donohue, Hemika Vempalli, Rebecca C. Thurston, Michele D. Levine, Katherine Snedaker, Kyla Z. Donnelly, David O. Okonkwo, Martina Anto-Ocrah

**Affiliations:** ^1^ Neuroscience, Kenneth P. Dietrich School of Arts & Science , University of Pittsburgh, Pittsburgh, Pennsylvania, USA.; ^2^ University of Pittsburgh School of Medicine, Pittsburgh, Pennsylvania, USA.; ^3^ Department of Medicine, Division of General Internal Medicine, University of Pittsburgh School of Medicine, Pittsburgh, Pennsylvania, USA.; ^4^ Department of Psychiatry, University of Pittsburgh School of Medicine, Pittsburgh, Pennsylvania, USA.; ^5^ PINK Concussions, Norwalk, Connecticut, USA.; ^6^ LoveYourBrain Foundation, Norwich, Vermont, USA.; ^7^ Department of Neurological Surgery, University of Pittsburgh Medical Center, Pittsburgh, Pennsylvania, USA.; ^8^ Department of Epidemiology Pittsburgh, University of Pittsburgh Graduate School of Public Health, Pittsburgh, Pennsylvania, USA.

**Keywords:** concussion, female, mild traumatic brain injury, mood, resilience, race

## Abstract

Resilience is associated with the degree to which post-concussion symptoms (PCS) are experienced. However, the role of resilience in the recovery trajectory of minority women, who tend to have prolonged concussion recovery, is poorly characterized. We evaluated the association between resilience and PCS, to determine if the association differed by race. A secondary data analysis was performed. Resilience was assessed using the Resilience Scale and PCS with the Rivermead questionnaire. Both variables were evaluated 6–10 weeks post-injury. Baseline demographics, spearman correlation, and multivariable linear regression models were used to determine the association between resilience and PCS. Seventy-seven women (mean age 28 ± 7.6) were included, 57% were White, and 43% were Black or Hispanic. The overall cohort had a moderate association between resilience and PCS (*R* = −0.304, *p* = 0.007). The association was present in minorities (*R* = −0.486, *p* = 0.004), and was stronger for Blacks (*R* = −0.745, *p* < 0.001). After adjusting for religion as a covariate separately, resilience (*β* = −0.156, 95% confidence interval [CI]: −0.285, −0.026; *p* = 0.019) and mood (*β* = 1.082, 95% CI: 0.847, 1.317; *p* < 0.001), were both independent predictors of PCS. The adjusted associations were stronger for the minority subgroup for both resilience (*β* = −0.231, 95% CI: −0.413, −0.050; *p* = 0.014) and mood (*β* = 1.122, 95% CI: 0.753, 1.491; *p* < 0.001). Our findings show that compared with Whites, minority individuals with higher resilience have greater resolution of PCS. However, mood is also of importance in this association. Thus resilience-based interventions must also target mood. Interventions that strengthen resilience may have promise in promoting equitable recovery in the setting of female concussions.

## Introduction

Annually, an estimated 42 million individuals globally experience a mild traumatic brain injury (mTBI), often called concussion.^1^ For many, the injury is associated with physical/somatic (i.e., headache, nausea, dizziness, fatigue, etc.), cognitive (i.e., memory recall, concentration, etc.), emotional/affective (i.e., depression, anxiety, irritability, etc.), and sleep disturbance (i.e., sleep deprivation, restlessness, etc.) symptoms that resolve within a few days or weeks post-injury.^2,3^ However, for 15–25% of patients, these symptoms may persist for at least 1 year post-injury.^4,5^ This group of patients, often referred to as the “miserable minority,” predominantly consists of women.^6^ Racial and ethnic minorities also tend to have delayed recovery.^7^

The intersection of gender, race, and health outcomes is starkly evident in concussion recovery. Compared with men, women report experiencing more pronounced cognitive deficiencies, a heavier physiological toll, and an extended duration of post-concussion symptoms (PCS) post-injury.^8–15^ For racial and ethnic minority patients, recovery outcomes are compounded by poorer access to care, lower referrals to rehabilitation services, less functional independence,^16^ and poorer employment outcomes^17^ following their head injuries^17^ compared with Whites.^7^ These disparities underscore the urgency of prioritizing the study of women and minority groups, as recommended by an executive order promoting the advancement of women’s health research^18^ and the National Institutes of Health.^19^ The focus on racial and ethnic minority women, who are less likely to have follow-up care post-concussion and consequentially poorer recovery outcomes is critical to these efforts.

Over the years clinical guidelines for concussion care have evolved, diverging from physical and mental rest to physical exercise and progressive return to activity protocols.^20^ However, access to such protocols for minority patients, is often hindered by the broader spectrum of social determinants of health (SDOH).^21^ True equity in concussion care necessitates addressing protective factors that promote minority patients’ recovery, despite the many adversities they may face after concussion. One such factor is psychological resilience. Psychological resilience is defined as the attitudes or beliefs that one can recover from adversity,^22^ and is one of several factors that may contribute to mTBI outcomes.^23–25^ High psychological resilience has been associated with less anxiety and depression symptoms, and lower PCS in mTBI patients; both in the acute and chronic injury phases.^26,27^ Given that this resilience can be taught and learned,^28^ understanding its role in the recovery trajectory of women and individuals belonging to racial/ethnic minority groups may provide personalized, patient-centered treatment alternatives for these subgroups of patients, to improve health equity outcomes after concussion.

In this study, we assess the association between resilience and PCS in female concussion patients, considering racial/ethnic differences. We hypothesized that higher resilience scores would be associated with lower PCS scores, and these associations would differ by race.

## Methods

This study was a secondary data analysis of a 2017 prospective cohort study examining the link between concussion and risk of sexual dysfunction in women of reproductive age.^29^

### Participants

The study population included women aged 18 to 45 years who received medical attention at a level 1 trauma center emergency department (ED) or related urgent care facility within seven days of their concussion. The definition of concussion adhered to by the Centers for Disease Control and Prevention and/or American Rehabilitation Medicine clinical definitions, and/or the diagnosis by an ED physician.^3,29^

### Procedures

The consent and the enrollment of patients were carried out by experienced and trained emergency medicine research associates in the ED.^29^ Written consent was obtained from all participants. The study received approval from the institutional review board at the University of Rochester.

### Measures

#### Predictor Value

Resilience was assessed with the resilience scale (RS),^30,31^ a commonly used resilience assessment. Participants were assessed using this scale 6–10 weeks post mTBI. The survey contains 25 items with the total scores of increasing resilience ranging from 25 to 175.^30^ This scale has been used widely in concussion research.^10,23,25,32^

#### Primary Outcome

##### PCS

The Rivermead PCS questionnaire (RPQ) was used to evaluate PCS outcomes. This questionnaire can be administered to patients acutely or later, to assess the patient’s concussion severity and recovery progress. RPQ evaluates 16 different symptoms commonly found after a concussion. Patients are asked to describe their symptoms in the last 24 h and to compare them to how severe these were before the concussion. These symptoms are reported by severity on a scale from 0 to 4: not experienced, no more of a problem, mild problem, moderate problem, and severe problem.^33^ Total scores range from 0 to 64, with lower scores indicative of reduced symptom burden and better recovery.

#### Covariates

A *priori* we considered the following demographic and concussion-related variables as important covariates to adjust for based on their association with PCS and/or resilience at the time of mTBI injury: age,^34,35^ education,^34^ history of concussion,^29^ injury mechanism,^29,34^ religiosity,^36,37^ and mood.^10,23–25,29,32,34^ All were self-reported except for mood, which was evaluated with the hospital anxiety and depression scale (HADS), a self-rating scale with two subscales measuring symptoms of depression (HADS-D) and anxiety (HADS-A). For each subscale, the range of scores is 0 to 21. A score ≥11 is considered a clinically significant disorder, whereas scores between 8 and 10 suggest mild anxiety or depressive disorders.^38,39^

### Statistical analyses

Chi-square and T-tests were used to evaluate the frequencies and distributions of the data stratified by race/ethnicity and PCS. Correlation coefficients were used to evaluate the association between resilience and PCS by race. Variables that were statistically significant in bivariate analyses (with PCS) were adjusted for multivariable analyses as confounders and/or predictors. Crude and adjusted linear regression models were fit to determine the effect estimates for the average change in PCS scores with each unit increase in resilience. We used *p* < 0.05 to determine statistical significance for all bivariate, crude, and adjusted analyses. All analyses were conducted using SPSS version 28.0.

## Results

Out of the 103 female concussion participants enrolled in the original study, 88 (78%) had follow-up data and 77 of the 88 (87.5%) had race/ethnicity data and were included in these secondary data analyses. The mean age of the sample was 28 ± 7.6; (range, 18–45 years). Over half (57%, *n* = 44) self-identified as White, and 43% (*n* = 33) identified as either Black (*n* = 21) or Hispanic (*n* = 12) and were categorized as minorities. Table 1 shows the demographic, psychosocial, and injury-related attributes of the 77 participants, stratified by race/ethnicity. The groups differed by education (Whites tended to have higher educational attainment [i.e., associate degree and higher] than minorities, 52.3% vs. 24.2% respectively; *p* = 0.01), and injury mechanism (assaults were more common for minorities: 4.5% White vs. 27.3% minority; falls were more common for Whites: 34.1% White vs. 9.1% minority; *p* = 0.01). There were no racial differences in resilience, PCS, religiosity, mood, or other correlates. In Table 2, we stratified the variables by PCS score to identify potential predictors to adjust for in regression analyses. Variables that met the *p* < 0.05 cutoff were resilience, religion, and mood (HADS-Total, HADS-A, HADS-D) scores.

**Table 1.** Demographic, Psychosocial, and Injury-Related Attributes of the Study Sample, Stratified by Race/Ethnicity (*n* = 77)

	Black or Hispanic (*n* = 33)	White (*n* = 44)	Total (*n* = 77)	*p*-value
Resilience scale [Mean (±SD)]	137.76 (±28.506)	128.36 (±22.854)	132.39 (±25.681)	0.113
Post-concussion symptom score (mean [±SD])	20.33 (±15.729)	21.23 (±14.802)	20.84 (±15.111)	0.799
Age (Mean [±SD])	28.15 (±6.751)	28.02 (±8.329)	28.08 (±7.645)	0.942
Higher education (*n* [%]) (Advanced/Terminal degrees)	8 (24.2%)	23 (52.3%)	31 (40.3%)	0.013
Religious (*n* [%])	16 (48.5%)	20 (45.5%)	36 (46.8%)	0.792
Relationship (*n* [%])				0.651
Single, no relationship	13 (39.4%)	13 (29.5%)	26 (33.8%)	
Relationship	13 (39.4%)	21 (47.7%)	34 (44.2%)	
Married/Divorced	7 (21.2%)	10 (22.7%)	17 (22.1%)	
With previous concussion history (*n* [%])	29 (87.9%)	33 (75.0%)	62 (80.5%)	0.158
Injury mechanism (*n* [%])				0.007
Assault	9 (27.3%)	2 (4.5%)	11 (14.3%)	
Fall	3 (9.1%)	15 (34.1%)	18 (23.4%)	
Motor-Vehicle/Motorcycle/Struck	16 (48.5%)	19 (43.2%)	35 (45.5%)	
Other	5 (15.2%)	8 (18.2%)	13 (16.9%)	
Hospital anxiety and depression scale clinical case (Score *≥* 11) (*n* [%])	11 (33.3%)	17 (38.6%)	28 (36.4%)	0.632

**Table 2.** Bivariate Comparison of Racial, Demographic, Psychosocial, and Injury-Related Attributes of the Study Participants, Stratified by Post-Concussion Symptoms (*n* = 77)

	PCS < 32 (*n* = 60)	PCS ≥ 32 (*n* = 17)	Total	*p*-value
Race/Ethnicity (*n* [%])				0.675
Black	15 (25.0%)	6 (35.3%)	21 (27.3%)	
Hispanic	10 (16.7%)	2 (11.8%)	12 (15.6%)	
White	35 (58.3%)	9 (52.9%)	44 (57.1%)	
Minority (Black or Hispanic) (*n* [%])	25 (41.7%)	8 (47.1%)	33 (42.9%)	0.692
Resilience scale (mean ([SD])^b^	136.58 (±24.492)	117.59 (±24.940)	132.39 (±25.681)	0.006
Age (mean [±SD])	27.85 (±8.128)	28.88 (±5.754)	28.08 (±7.645)	0.626
Higher education (*n* [%]) (Advanced/Terminal degrees)	24 (40.0%)	7 (41.2%)	31 (40.3%)	0.930
Religious (*n* [%])	32 (53.3%)	4 (23.5%)	36 (46.8%)	0.030^a^
Relationship (*n* [%])				0.535
Singe, no relationship	22 (36.7%)	4 (23.5%)	26 (33.8%)	
Relationship	26 (43.3%)	8 (47.1%)	34 (44.2%)	
Married/Divorced	12 (20.0%)	5 (29.4%)	17 (22.1%)	
With previous concussion history (*n* [%])	50 (83.3%)	12 (70.6%)	62 (80.5%)	0.242
Injury mechanism (*n* [%])				0.431
Assault	10 (16.7%)	1 (5.9%)	11 (14.3%)	
Fall	12 (20.0%)	6 (35.3%)	18 (23.4%)	
Motor-Vehicle/Motorcycle/struck	27 (45.0%)	8 (47.1%)	35 (45.5%)	
Other	11 (18.3%)	2 (11.8%)	13 (16.9%)	
HADS total score^a^	10.38 (±7.042)	26.18 (±10.126)	13.87 (±10.177)	< 0.001^a^
HADS anxiety score^a^	5.75 (±4.845)	13.94 (±5.717)	7.56 (±6.067)	< 0.001^a^
HADS depression score^a^	4.63 (±2.905)	12.24 (±4.893)	6.31 (±4.655)	< 0.001^a^
HADS clinical case (score *≥* 11) (*n*, %)	14 (23.3%)	14 (82.4%)	28 (36.4%)	< 0.001^a^

^a^ Included in bivariate analyses as HADS referring to mood.

^b^ Independent variable of interest. HADS, hospital anxiety and depression scale.

**FIG. 1.**
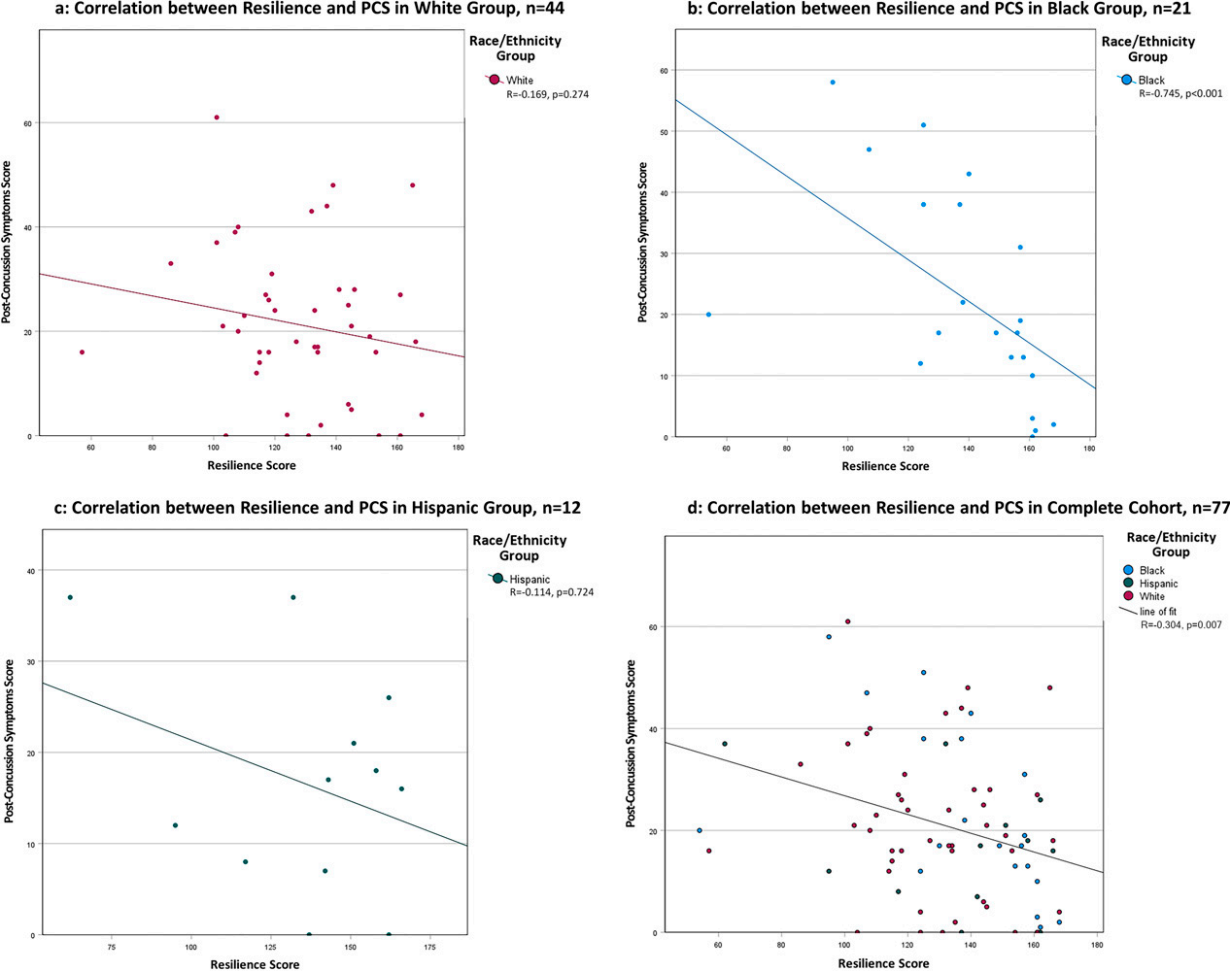
This figure comprises four scatter plots representing the correlation between resilience and post-concussion symptoms scores across different racial/ethnic groups and the complete cohort. Data points and trendlines in red refer to the White group, blue for the Black group, green for the Hispanic group, and Black for the entire cohort. **(a)**: Correlation in the White group (*n* = 44), with a correlation coefficient *r* of −0.169 and a *p*-value of 0.274. **(b)**: Correlation in the Black group (*n* = 21), with *r* = −0.745 and *p* < 0.001. **(c)**: Correlation in the Hispanic group (*n* = 12), with *r* = −0.114 and *p* = 0.724. **(d)**: Correlation in the complete cohort (*n* = 77), with *r* = −0.304 and *p* = 0.007.

As shown in Fig. 1, the correlation between resilience and PCS for Whites was low and not statistically significant (Fig. 1a, *n* = 44, *R* = −0.169 [95% confidence interval [CI]: −0.451, 0.144], *p* = 0.274) but stronger for the minority group (*n* = 33, *R* = −0.486 [95% CI: −0.716, −0.161], *p* = 0.004); particularly for Blacks (Fig. 1b, *n* = 21, *R* = −0.745 [95% CI: −0.893, −0.452], *p* < 0.001). There were no significant findings for Hispanics (Fig. 1c, *n* = 12, *R* = −0.114 [95% CI: −0.657, 0.506], *p* = 0.724).

In multivariate linear regression models, crude models showed an average decrease of −0.184 in PCS scores with increasing resilience (Table 3). Adjustment for religion accounted for some but not all of the association between resilience and PCS scores (Table 4). Given psychological resilience’s association with mood disorders,^40^ the interaction term between resilience and mood was also included to assess whether the relationship between mood symptoms and PCS differs depending on levels of resilience. The addition of mood to models, however, markedly changed the direction of the effects estimates, although interaction terms (mood and resilience) were not statistically significant (Table 4). Although affected by collinearity, it was still important to model the interaction between predictors (Table 5) to observe outstanding conditional relationships, however, none were statistically significant. We therefore modeled resilience and mood independently and found that the Black group showed the strongest association between resilience and PCS scores (Table 6, *B* = −0.341, *p* = 0.010), as well as between mood and PCS scores (Table 6, *B* = 1.231, *p* < 0.001). For Whites, resilience was not significantly associated with PCS scores (Table 6, *B* = −0.115, *p* = 0.250), but the negative mood was significantly associated with greater PCS scores (Table 6, *B* = 1.084, *p* < 0.001). No associations were statistically significant for the Hispanic group (Table 6).

**Table 3.** Crude Unadjusted Multivariate Linear Regression Models Predicting the Association with Post-Concussion Symptom Scores (*n* = 77)

Model	Variable	B	Beta	t	95% CI	*p*-value
Resilience	Resilience	−0.184	−0.313	−2.852	−0.313, −0.055	0.006
Mood (HADS)	HADS	1.113	0.749	9.797	0.886, 1.339	<0.001
Race/Ethnicity	Race/Ethnicity	−0.300	−0.017	−0.150	−4.274, 3.674	0.881

**Table 4.** Adjusted Multivariate Linear Regression Models Predicting the Association with Post-Concussion Symptom Scores (*n* = 77)

Model	Variable	B	Beta	T	95% CI	*p*-value	VIF
Religious-Adjusted resilience	Resilience	−0.156	−0.264	−2.389	−0.285, −0.026	0.019	1.055
	Religious	6.401	0.213	1.923	−0.233, 13.036	0.058	
Religious-Adjusted mood (HADS)	HADS	1.082	0.728	9.172	0.847, 1.317	<0.001	1.078
	Religious	2.323	0.077	0.972	−2.438, 7.085	0.334	
Resilience-Adjusted Race/Ethnicity	Race/Ethnicity	−1.346	−0.078	−0.696	−5.202, 2.510	0.489	1.035
	Resilience	−0.193	−0.327	−2.921	−0.324, −0.061	0.005	
Mood (HADS) — Adjusted Race/Ethnicity	Race/Ethnicity	−0.185	−0.011	−0.139	−2.835, 2.466	0.890	1.000
	HADS	1.112	0.749	9.731	0.885, 1.340	<0.001	
Resilience and Mood (HADS) -Adjusted resilience	Resilience	0.024	0.041	0.478	−0.077, 0.126	0.634	1.273
	Religious	2.467	0.082	1.019	−2.358, 7.292	0.312	1.095
	HADS	1.108	0.746	8.502	0.848, 1.367	<0.001	1.301

**Table 5.** Interaction Multivariate Linear Regression Models Predicting the Association with Post-Concussion Symptom Scores (*n* = 77)

Model	Variable	B	Beta	T	95% CI	*p*-value	VIF
Resilience and mood (HADS) interaction model	Resilience	0.029	0.049	0.327	−0.146, 0.204	0.744	3.719
	HADS	1.229	0.828	1.892	−0.065, 2.524	0.062	31.905
	Resilience*HADS	−0.001	−0.060	−0.151	−0.011, 0.009	0.880	26.295
Race/Ethnicity and resilience interaction model	Race/Ethnicity	−14.970	−0.866	−1.462	−35.376, 5.437	0.148	29.320
	Resilience	−0.411	−0.698	−2.362	−0.757, −0.064	0.021	7.294
	Race/Ethnicity*Resilience	0.102	0.820	1.355	−0.048, 0.251	0.180	30.617
Race/Ethnicity and Mood (HADS) interaction model	Race/Ethnicity	0.791	0.046	0.351	−3.697, 5.279	0.726	2.838
	HADS	1.258	0.847	4.291	0.673, 1.842	<0.001	6.512
	Race/Ethnicity*HADS	−0.066	−0.120	−0.538	−0.312, 0.179	0.592	8.293

**Table 6.** Multivariate Linear Regression Models Predicting the Association Between Resilience and Post-Concussion Symptom Scores Stratified by Race

	Model	B	Beta	t	95% CI	*p*-value	VIF
Effect estimates of association with RPQ stratified by race, White only (*n* = 44)	Crude Resilience	−0.115	−0.177	−1.167	−0.313, 0.084	0.250	—
	Religious-Adjusted Resilience	−0.070	−0.107	−0.689	−0.274, 0.134	0.495	1.091
	Crude Mood	1.084	0.733	6.978	0.771, 1.398	<0.001	—
	Religious-Adjusted Mood	1.050	0.710	6.500	0.724, 1.376	<0.001	1.073
Effect Estimates of association with RPQ stratified by Race, Minorities (*n* = 33)	Crude Resilience	−0.249	−0.452	−2.822	−0.430, −0.069	0.008	—
	Religious-Adjusted Resilience	−0.231	−0.419	−2.607	−0.413, −0.050	0.014	1.027
	Crude Mood	1.148	0.769	6.690	0.798, 1.499	<0.001	—
	Religious-Adjusted Mood	1.122	0.751	6.208	0.753, 1.491	<0.001	1.083
Effect Estimates of association with RPQ stratified by race, Black (*n* = 21)	Crude Resilience	−0.341	−0.546	−2.839	−0.592, −0.089	0.010	—
	Religious-Adjusted Resilience	−0.321	−0.515	−2.567	−0.584, −0.058	0.019	1.057
	Crude Mood	1.231	0.844	6.854	0.855, 1.606	<0.001	—
	Religious-Adjusted Mood	1.204	0.826	6.508	0.815, 1.593	<0.001	1.038
Effect Estimates of association with RPQ stratified by race, Hispanic (*n* = 12)	Crude Resilience	−0.134	−0.337	−1.130	−0.399, 0.130	0.285	—
	Religious-Adjusted Resilience	−0.127	−0.318	−1.042	−0.402, 0.148	0.324	1.006
	Crude mood	0.718	0.409	1.418	−0.410, 1.845	0.186	—
	Religious-Adjusted Mood	0.647	0.369	1.078	−0.711, 2.005	0.309	1.276

RPQ, Rivermead Post-Concussion questionnaire.

## Discussion

Our results show that resilience may play a role in the recovery of female concussion patients, particularly patients who self-identified as Black. However, there may be other factors that mediate the relationship between resilience and recovery, therefore longitudinal data are needed to address questions about the nature of the association between resilience and recovery. One such factor is mood; it also plays an unignorable and important role in concussion recovery and cannot be overlooked.

Although resilience is crucial for individuals of all racial and ethnic backgrounds following TBIs, recent research suggests that individuals belonging to racial or ethnic minority groups, though not differing in our study, may possess higher levels of resilience compared with White populations due to factors such as social cohesion and a strong sense of community.^41^ However, this does correlate with our findings that resilience is associated with recovery for minorities, who may need to rely more heavily on resilience due to navigating a greater number of stressors related to SDOH.^21^ The need for resilience among African Americans has been shaped by enduring systemic challenges and adversities, such as racial discrimination and socioeconomic disparities, which may necessitate a greater reliance on resilience in navigating the health care system. This psychological resilience has been a means of survival for African Americans, allowing them to navigate and overcome obstacles throughout American history.^42,43^ Hispanic populations in the United States have also needed to develop resiliency over time, through family cohesion, intergenerational support, and cultural values that emphasize community and collective well-being.^44^ These strengths have historically enabled Hispanic communities to withstand social and economic hardships as well.^44^

In the context of health and recovery, studies suggest that religion is often a source of resilience and support through spirituality and religious communities.^45^ In both African American and Hispanic communities, it is common for the church and religiosity to play a pivotal role in fostering a sense of hope and collective strength, contributing to better mental health outcomes and resilience in the face of illnesses.^36,37^

Conversely, White populations may not exhibit the same level of resilience due to different historical experiences and societal positions. Trust in the healthcare system and access to resources can influence health outcomes and the necessity for resilience. White individuals may have more trust in the health care system and are more likely to receive follow-up care, which could impact their reliance on factors such as resilience when recovering from health conditions.^46^

In relation to mood in the recovery from PCS, its significance has also been corroborated by other studies. Mood disorders, particularly anxiety and depression, have been identified as key factors that can influence the duration and severity of PCS. For instance, Narducci et al. found that mood screening scores (PHQ-9 and GAD-7) were predictive of concussion recovery,^47^ and other papers found an association between psychiatric symptoms and patients experiencing persistent PCS.^48,49^ These findings align with the findings of our paper, suggesting that mood is a factor in concussion recovery across all racial/ethnic groups.

Understanding the universal importance of mood in concussion recovery across racial and ethnic groups of individuals is crucial, as it underscores the need for tailored interventions that address the unique challenges faced by minority populations. Implementing resilience-building programs within the ED setting, where most concussion patients initially seek care, as in this cohort, maybe the pivotal step needed to enhance recovery outcomes for concussion patients. A triage system could be developed to assess resilience at intake. Based on this assessment, a brief “resilience boost” could be delivered during the ED visit, such as through a short educational session or the provision of resilience resources such as information on coping strategies, all tailored to fit within the ED’s time constraints. If effective, interventions of this nature could delay the compounding effects of mTBI for minority patient populations, who tend to be younger, unemployed, unmarried, of lower socio-economic status, and mired by multiple SDOH, as discussed earlier.^50–52^ Some may argue that a busy ED may not be the ideal setting for such a screening and intervention program, however, emerging research shows a surge in ED-based women’s health screening and intervention programs. Programs for cervical cancer,^21^ domestic violence, and others, that do not interfere with patients’ care (including length of stay), have already been successfully delivered, are favored by ED directors,^53^ and address SDOH.^54,55^

Resilience assessment and resilience-boosting interventions can be implemented by primary care physicians, concussion clinics, and neurology departments, which would provide a pivotal role in ongoing management and coordination of care as these places are the secondary tier of support after ED. Also, integrating resilience assessment in the standard post-concussion assessment^56^ could improve healthcare providers’ ability to incorporate resilience development into patient care strategies. Interventions of this nature would be additionally beneficial for minority groups who tend to use the ED as their usual place of care^57^ and due to societal adversity, are more likely to use complementary health approaches, such as prayer and healers often resilience boosting, as part of their treatment.^58^ By integrating these culturally relevant practices into hospital-based interventions, health care providers can offer a comprehensive resilience boost that empowers patients to navigate the complex landscape of SDOH^21^ upon discharge.

Pre-discharge workshops that introduce patients to resilience-building techniques and resources would be beneficial. Kreutzer et al. suggest a Resilience and Adjustment Intervention (RAI) which includes education, skill building, and psychological support after assessing its impact with a randomized control trial. After seven 1-h sessions (over 5 weeks), the authors report that exposed participants had a statistically significant increase in resilience and reduced psychological distress^59^ compared with the control group. In addition to such an intervention, collaboration with social workers to address further SDOH barriers such as insurance coverage for specialty/referral services, transportation, income, and access to follow-up care (to name a few). Lastly, before discharge, referrals to outpatient resilience programs such as LoveYourBrain, which provides holistic, community-based rehabilitation services, have also been proven to be fruitful.^60^ Peer support programs such as PINK Concussions,^61^ the first non-profit organization to focus on pre-injury education and post-injury medical care for women and girls with brain injury; as well as several psychosocial support systems for addressing systemic barriers to care for racial/ethnic minorities^61^ may also be recommended.

Despite its novelty, our study has some limitations that bear mentioning. First, the modest sample sizes, and therefore power, particularly among Hispanic participants, underscore the importance of conducting larger and more representative studies to further broaden our understanding of resilience’s role across diverse racial/ethnic groups to see if resilience’s effect is specific to the Black population only or all racial/ethnic minority groups. Secondly, longitudinal assessments are needed to fully capture the evolution and longer-term impacts of resilience on concussion recovery. Finally, despite women having more protracted concussion recovery, expanding the research to include men, and other sex/gender minorities may enhance the impact of the study findings. Despite these limitations, our findings illuminate a path forward for research aimed at eliminating health disparities and fortifying protective factors.

## Abbreviations Used

ED: Emergency Department
EDRAs: Emergency Medicine Research Associates
GAD-7: General Anxiety Disorder-7
HADS: Hospital Anxiety and Depression Scale
HADS-D: Hospital Anxiety and Depression Scale, Depression Subscale
HADSA: Hospital Anxiety and Depression Scale, Anxiety Subscale
PHQ-9: Patient Health Questionnaire-9
RAI: Resilience and Adjustment Intervention
RPQ: Rivermead Post-concussion Symptoms Questionnaire
RS: Resilience Scale
SDOH: Social Determinants of Health
VIFs: Variance Inflation Factors
