# Supplementary material for: Resilience and Concussion Recovery in Minority Women: Promoting Health Equity
Source: Neurotrauma Rep. 2024 Oct 9;5(1):989–97. doi: 10.1089/neur.2024.0075 (PMC11491579; doi:10.1089/neur.2024.0075)
Supplement: Supplementary Table S1 [file neur.2024.0075_supp_tables1.pdf]

Table 7: Zero-Order Correlation Matrix

	Resilience	RPQ	Race/Ethnicity	HADS
Resilience	1	-	-	-
Post-Concussion Symptoms Score (RPQ)	-0.304*<br>(95% CI: -0.499, -0.079)<br>p=0.007	1	-	-
Race/Ethnicity	-0.251*<br>(95% CI: -0.455, -0.022)<br>p=0.028	0.032<br>(95% CI: -0.200, 0.260)<br>p=0.785	1	-
Mood (HADS)	-0.504*<br>(95% CI: -0.658, -0.309)<br>P<0.001	0.695*<br>(95% CI: 0.553, 0.798)<br>p<0.001	0.032<br>(95% CI: -0.200, 0.260)<br>p=0.785	1
